# Benefits and barriers: a qualitative study on online social participation among widowed older adults in Southwest China

**DOI:** 10.1186/s12877-021-02381-w

**Published:** 2021-08-03

**Authors:** Yan Hong, Jingjing Fu, Dehui Kong, Siqi Liu, Zhu Zhong, Jing Tan, Yu Luo

**Affiliations:** School of Nursing, Army Medical University / Third Military Medical University, No. 30 Gaotanyan Street, Shapingba District, Chongqing, 400038 People’s Republic of China

**Keywords:** Widowhood, Older adults, Social participation, Online activities, Qualitative research

## Abstract

**Background:**

With the development of digital media, online activities are increasingly becoming part of the daily life of older adults. Widowed older adults generally would face changes in social interactions and activities due to widowhood; thus, the importance of online participation may be more prominent in this population. However, a detailed evidence on the experiences of online social participation among widowed older adults is relatively sparse. This study aimed to explore widowed older adults’ perceptions regarding online social participation in southwestern China.

**Methods:**

This study adopted a qualitative approach. Semi-structured, in-depth individual interviews were conducted with 19 widowed older adults between September–December 2020. Thematic analysis was applied to analyse the data.

**Results:**

Two major themes, “benefits” and “barriers” were identified from the original data analysis. Subcategories concerning the theme “benefits” were “benefit perception (convenience, flexible time, supplementation)”, “health promotion”, “emotional comfort”, and “social connection”. Subcategories of “barriers” were “worries: personal economic loss”, “concerns: security of digital device”, “troubles: the diversity of online social participation”, and “difficulties: using digital media”.

**Conclusions:**

Social participation of widowed older adults in southwestern China has begun to be integrated into the digital world; however, it remains at an early stage with the simple purpose of engagement. The older adults may face many challenges for online social participation. Although there are barriers and challenges in online social participation, widowed older adults can reap its benefits, which can be used as an important measure to facilitate a fulfilling life and successful ageing. There is no doubt that online social participation will become a trend within the foreseeable future. Family, friends and health care professionals should pay more attention to the needs of online social participation in widowed older adults and provide adequate support for them to achieve a meaningful life.

## Background

In recent times, the acceleration of ageing population poses a huge challenge for the entire world. In developed countries, the term ‘older adults’ typically refers to people over 65 years old [1]. In developing countries, the ‘older adults’ typically refers to people over 60 years old since these countries have a lower overall life expectancy [2]. Thus, people over the age of 60 were defined as older adults in our study. It is predicted that, by 2050, the number of people over the age of 60 would increase to 2.1 billion [3], and China’s older population could reach 480 million [4]. As widowhood is a common event in the lives of older adults, the rapid growth of older population indicates that the number of widowed older adults will inevitably increase dramatically. Widowed older adults are likely to face major life changes, such as social isolation [5], loss of financial security [6], changes in leisure activities [7], and many stressful life events [8].

Aroogh and Shahboulaghi further analysed the social participation of older adults using the Walker and Avant method of concept analysis [9]. Referring to their concept, in our study, we defined social participation as a conscious and active engagement in various social activities leading to interacting and sharing resources with other people directly or indirectly, including leisure activities, entertainment, social interaction, and volunteering. However, the common activities related to older adults’ social participation include meeting friends, exercising, volunteering, church activities, and participating in hobby clubs [10–13].

The rapid advancement of digital media - personal computers, tablets, smartphones, and social media – helps engage people in online activities in their daily lives [14]. For example, listening to music and watching videos, making new friends, shopping, connecting with others, visiting a forum, and ordering food can all be accomplished with the help of digital media in today’s day and age [15, 16]. Additionally, digital media have been found to be an efficient means to promote social participation for older adults [14, 17–19]. Therefore, digital media provides older adults with more life choices and opportunities for social participation, thereby ensuring a fulfilling life.

Participation is a significant embodiment of active ageing. It has been found that promoting social participation helps older adults achieve successful ageing [20]. The World Health Organization has proposed that social participation is a key policy to cope with the ageing of global population [21]. Activity theory believes that social participation can have a positive impact on the older population. Several studies have documented that social participation could slow down the functional decline in older adults and reduce mortality and morbidity [22, 23]. Furthermore, gardening, meeting friends, volunteering, and application of new technologies were found to reduce the feeling of loneliness in older adults [24, 25]. Matz-Costa et al. [26] found that widowed older adults participated in several socially purposeful activities, including physical activity, social interaction, and emotional exchange, which not only promoted healthy behaviour but also increased their self-esteem, life control, and life meaning, thereby helping them relieve the grief caused by widowhood. Furthermore, online activities help the older adults to think more actively and improve their cognitive function [27]. To summarize, social participation has a positive effect on the physical and mental health and social adaptation of older adults.

Currently, the overall level of online social participation of older adults is rising. However, owing to an unbalanced social and economic development, differences in the depth and breadth of online social participation for older adults exist in different regions. Prior studies mainly focussed on the patterns of social participation and its influencing factors [7, 13, 28], the relationship between social participation and health [29, 30], and the role of digital media in social participation [14]. However, there was a dearth of qualitative studies on online social participation among widowed older adults. Therefore, this phenomenological study aimed to explore widowed older adults’ perceptions regarding online social participation using in-depth interviews.

## Methods

### Study design

A qualitative design was adopted in this study, which allowed for an in-depth insight into the views about online social participation among widowed older adults. This qualitative study followed the Consolidated Criteria for Reporting Qualitative Research (COREQ) [31].

### Settings and participants

Purposive sampling was used to recruit participants from three teaching cooperative communities in Chongqing, a big city in southwestern China. In the city, the population of those aged over 60 was 7.1955 million, accounting for 21.13% of the total population [32], which was higher than the average level in China. Information about the study was first sent to the teaching cooperative communities. Thereafter, the staff of the community health service centre helped identify potential participants and provide them with an information sheet. The older adults contacted the researcher directly or via the staff of the community health service centre if they wished to participate in the study. The inclusion criteria for the participants were as follows: (1) aged between 60 and 90 years-old; (2) widowed, without a remarriage or dating post widowhood; (3) those who could understand the questions and express himself/herself clearly; and (4) those willing to participate in the study. The sample size in qualitative studies depends on whether required information is saturated [33]. The recruitment process of participants continued simultaneously with the collection of data, which were analysed until saturation was reached. After interviewing 19 participants, no new information could be obtained from interviewing the rest of the participants. To further verify whether the saturation was truly reached, we continued to investigate two additional participants and found that the information was indeed saturated. Therefore, we only analysed the data of the first 19 interviewees.

### Data collection

A set of interview questions were originally designed based on the purpose of the study and literature review. They were then appropriately modified according to the experts’ opinions and pre-test result. Some examples of specific questions are listed as follows: (a) ‘Nowadays, some older people use digital media (e.g. personal computer, tablet computers, and smart phone) to participate in various online activities, including social networking, shopping, seeking information, and health-related activities. Have you ever participated in such activities? What activities have you participated in? Please describe them in detail.’; (b) ‘How do you feel about online social participation?’; (c) ‘What changes have online activities brought to your life?’; and (d) ‘What difficulties did you encounter during the participation?’

Individual in-depth interviews were conducted to collect participants’ information. The time and setting of interviews were determined by the participants. All interviews were conducted in a quiet room (two participants were in their own home due to impaired mobility, and others were in the office provided by the community) by two researchers, both of whom were doctoral degree candidates and experienced in conducting qualitative studies. Informed consent was obtained after explaining the purposes and the contents of the study before the interview. Prior to being interviewed, participants were required to complete a brief demographic questionnaire. Digital recording was used throughout the interview process to ensure the accuracy and reliability of data. Meanwhile, the non-verbal behaviour of the participants was also observed and recorded. Each interview lasted approximately 30–50 min. This study’s data were collected from September to December 2020.

### Data analyses

Data collection and analysis were conducted concurrently. Initially, the audio recordings were transcribed verbatim. For accuracy, the researchers repeatedly listened to the audio files and read the transcriptions. Thereafter, thematic analysis was applied to analyse the data, a method to identify, analyse, and report themes within data and describe patterns across qualitative data, which was developed by Braun and Clark [34].

The analysis was done manually. First, two researchers (YH and JF) separately read the full text carefully and repeatedly to get familiarised with the data. They met to share and discuss their overall understandings of the data to determine the basic meanings. Second, they separately noted initial codes on the transcripts. Third, to search for themes, they separately categorised different codes into potential themes and sorted out all relevant central quotations. Fourth, JT and YL reviewed the themes. In a face-to-face meeting, they compared the findings of the above two researchers and clarified the similarities and differences between the researchers’ answers on the same topics, and finally reached an agreement on the themes through a discussion. Fifth, they defined and named themes through further reflections and discussions. Finally, YH initiated writing the thematic findings to produce a report, and the other authors provided continuous commentary on the writing in process.

To ensure trustworthiness of the study, the credibility, transferability, dependability and confirmability have to be taken into consideration [35]. We achieved credibility through the following measures. First, the selection of participants, data collection and analysis were conducted as carefully as we managed, to enhance credibility of the study. Second, four authors were actively took part in the processes of data analysis and interpretation and were aware of our preunderstandings and our influence on the emerging findings. Third, regular meetings were held to discuss the data, codes, and themes. Finally, to verify the authenticity of the results, researchers checked with the participants and discussed the findings with them. The results can be transferred to other contexts or settings, which was achieved by providing an detailed description of the participants and the research process. To increase dependability, the transcripts were reviewed several times and then coded by two authors. Interpretations were also based on consensus among the authors. Confirmability concerns the aspect of neutrality. The interpretation should not be based on researchers’ own particular preferences and viewpoints but needs to be grounded in the data. To ensure confirmability, we have illustrated the themes by using quotations assigned with a code that refers to the participants who made the statements.

## Results

A total of 19 widowed older adults with a mean age of 73.84 years (range 64–87 years) were interviewed. Of them, 12 (63.16%) were women, seven (36.84%) were men, and 13 (68.42%) lived alone. Fifteen participants (78.95%) had chronic diseases - including hypertension, coronary heart disease, cervical spondylosis, diabetes, osteoporosis, Parkinson’s disease, cerebral infarction, epilepsy, and hyperlipidaemia - which would not affect their cognition. The detailed characteristics of the participants are shown in Table 1.

**Table 1:** Demographic characteristics of the participants

Variables	
**Age**	
60–69	6
70–79	8
80–89	5
**Gender**	
Male	7
Female	12
**Education**	
Illiterate	1
Primary school	4
Junior high	10
Senior high	3
College	1
**Time widowed (years)**	
≤ 1	4
2–4	6
≥ 5	9
**Type of residence**	
Living alone	13
Living with children	5
Living with grandchildren	1
**Monthly income (CNY)**	
1000–2000	4
2001–3999	12
≥ 4000	3
**Pension source**	
Retirement allowance	15
Commercial insurance	3
Funds by adult children	1
**Number of chronic diseases**	
None	4
1–2	13
≥ 3	2

**Fig. 1:**
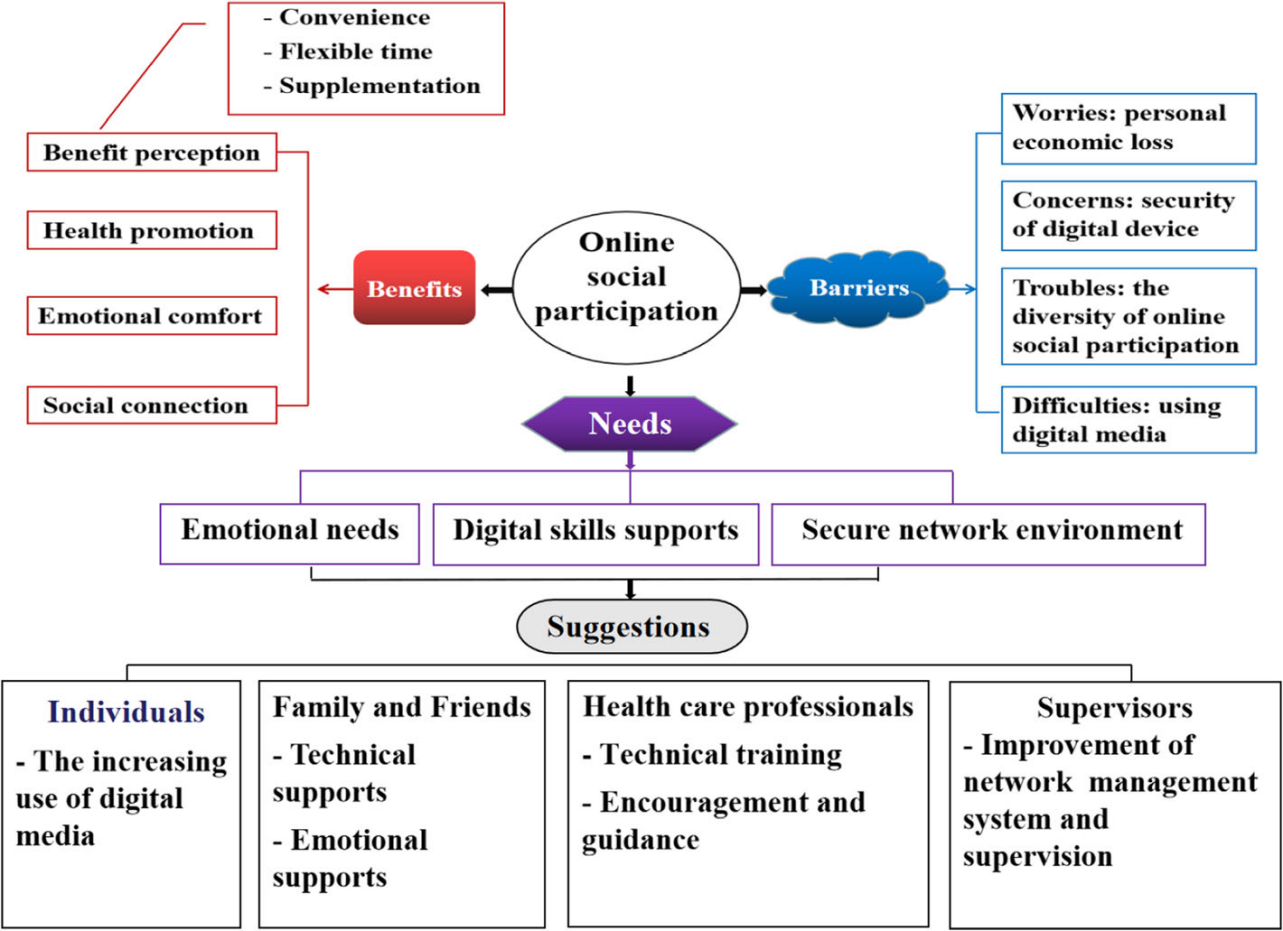
Domains of benefits and barriers and proposed suggestions about online social participation

Data analysis revealed two major themes: benefits (benefit perception, health promotion, emotional comfort, and social connection) and barriers (worries: personal economic loss, concerns: security of digital device, troubles: the diversity of online social participation, and difficulties: using digital media). The domains of the themes are presented in detail in Fig. 1.

### Benefits

#### Benefit perception

Almost all participants could perceive the advantages of online social participation, including convenience, flexible time, and supplementation.

The first benefit is convenience. Most participants reported that they could get involved in online activities anytime and anywhere.

*‘I mainly use mobile chat application to communicate with family and friends. I can chat with distant relatives and friends sitting in my own home. I find that communication between people has become easier ... We can see each other through a video chat, which is convenient for knowing the current situation...’ (D)*

*‘I like all kinds of online activities, such as social networking, shopping, and online learning, which provide great convenience.’ (N)*

Flexible time is another advantage of engaging in online activities. Some interviewees reported that they did not have time to get involved in some offline activities with friends because they had to help their children with household work or take care of their grandchildren. However, they could use their spare time to participate in online activities.

*‘I feel happier when I go outside with friends. I am willing to participate in outdoor activities, but I do not have time to do so. I have to look after my youngest grandson ... If I am free at home, I would contact someone else through the mobile chat app, as well as watch videos, or browse news and health information.’ (G)*

Supplementation is also an important advantage. Some interviewees described that age-related changes in physical health and function could increase their vulnerability, which would limit their participation in offline activities. Meanwhile, the outbreak of the pandemic also led to the limitation of offline social participation. However, to a certain extent, online activities can make up for the insufficient offline social participation. Older adults can rely on the Internet and smart phones to participate in online activities without having to go out.

*‘I have rarely been out since I was diagnosed with Parkinson’s 2 years ago ... I like chatting with my friends on the phone.’ (I).*

*‘Because of the outbreak of COVID-19, I could not go out and meet my friends and relatives, and only interacted with them online.’ (D).*

#### Health promotion

Online activities help promote healthy behaviours. With advancing age, older adults begin to pay attention to information on health promotion and disease prevention, such as diet and nutrition, exercise, and medication.

*‘I often pay attention to health information on my phone. I cannot ignore that knowledge, because I need to know how to take medicine and how to eat healthier.’ (N).*

Several interviewees talked about how they would exercise according to the fitness videos sent to their phone by other people.

*‘I follow the fitness videos sent by relatives or friends to exercise.’ (M).*

#### Emotional comfort

Widowed older adults are often susceptible to experiencing negative emotions such as grief, loneliness, depression, and exclusion, as well as feeling socially isolated. However, they may receive emotional comfort through online social participation.

Interviewees talked about how they felt happy when they contacted their family and friends through messages, voice chat, or video chat.

*‘I often video chat with my family at night, and I feel happy when I see my grandson.’ (G).*

Most participants used online activities help to relieve loneliness, as they experienced acute loneliness after being widowed, especially those who lived alone.

*‘I lived alone. When I felt lonely at home, I would watch some interesting videos, and chat with friends on my phone. Additionally, I would pay attention to the life that my friends shared on social media and write some comments on their posts.’ (R).*

#### Social connection

Social networking can also facilitate contacts with relatives and friends. The interviewees stated that they frequently interacted with family and friends through mobile chat applications. They often shared articles, pictures, news, or videos with relatives and friends.

*‘I like contacting with my close friends. I often actively send them articles, which are meaningful, humorous, or educational, and they also send such articles to me.’ (N).*

More than half of the participants described that they faced difficulties in using smartphones and corresponding mobile applications at first, but they would seek help from relatives and friends to learn about using their smartphones and applications, which would also promote a connection with them.

*‘I did not know how to use it at first, then my daughter taught me, and I was glad that she did. Now I face no problems in using it.’ (N).*

*‘When I did not know how to operate, I asked for help from my friends. They taught me how to use the chat application.’ (M).*

### Barriers

#### Worries: personal economic loss

Older people generally worry about economic losses stemming from both personal and external reasons.

Visual degradation is common among older adults, which reduces their acceptance and openness towards online activities. In this study, it was found that some interviewees with poor eyesight do not indulge in online shopping for the fear of economic loss due to mistake in operation.

*‘I do not like online shopping as I have poor eyesight, and I am worried about making mistakes.’ (D).*

Online shopping platforms generally require personal information of the customers. However, some Trojan software, Internet interception, and malicious cyber-attack can snatch people’s payment passwords while shopping online. Moreover, the unending fake websites and false information, along with new frauds taking place in succession will increase potential risks of online payment security. This was confirmed by most of our interviewees, as they were concerned about being cheated during online shopping:

*‘I would definitely not shop online, as it is increasingly difficult to be vigilant online as we age. There are so many cheaters on the Internet, and I am a little bit worried.’ (M).*

*‘I do not want to pay for something with my phone, because I am worried that others will know my bank card number and steal my money.’ (Q).*

#### Concerns: security of digital device

In this study, several widowed older adults expressed their concerns about the safety of their digital devices, which included devices like smartphones, computers, and digital TVs:

*‘I have read a piece of news about a phone exploding, so I am worried that the smartphone will explode while charging.’ (M).*

#### Troubles: the diversity of online social participation

Driven by the wave of global information, people have been woven into the entire social network, which not only meets the basic physiological needs, such as reservation service and shopping, but also helps achieve self-value, including economic, cultural, and political participation. However, the participants described that diversified forms of online social participation also bring confusion and trouble.

*‘There are many activities I can take part in through smartphones. However, these diversified activities have confused me. Owing to my advanced age, I do not have a good eyesight and enough energy now, and I do not know which activities are suitable for me. Therefore, I cannot get involved in all the activities, and I just choose some of them that I am interested in and can benefit from.’ (N).*

#### Difficulties: using digital media

New digital media has increasingly attracted the younger generation; however, the concept remains new to the older population. Therefore, most participants reported difficulties in using digital media and lacked self confidence in using them.

*‘I just know how to do simple operations on a smartphone; of course, it is difficult for me to do complicated operations on it. For example, I do not know how to complete the payment when shopping online.’ (P).*

*‘We are old and do not know how to use these, as we do not have prior knowledge. You know, it is not easy for us to use smartphones and computers. We are afraid of making mistakes.’ (Q).*

## Discussion

This study demonstrates that online social participation is a form of social participation that is worth exploring for older adults owing to its multi-faceted benefits. First, online social participation is based on the development of digital media; consequently, it is a part of social participation. It has the advantage of digital media, with continuous communication unbounded by time or place [36]. Therefore, it is beneficial for widowed older adults to participate in online activities anytime and connect with people across larger geographic distances. Even the widowed older adults with busy housework can participate in online activities in their free time at home. In particular, online social participation can be used in lieu of social participation for disabled and socially isolated older adults [37, 38], our results also supports this view. Second, it stimulates a healthy behaviour among the older adults. In our study, some participants focussed on health-related information available online. This is also in line with a previous study that found that some older people search for health information or consult a doctor online [19]. Moreover, some interviewees liked exercising according to fitness videos sent by others. Owari et al. reported that physical exercise is closely related to alleviation of mental distress and better physical performance [30]. It is noteworthy that older adults participate in various online activities, such as information searching, shopping, and economic management, which also stimulate brain activity and help improve cognition [39]. Third, online activities help widowed older adults receive emotional comfort. The participants of this study reported that they had no one to chat with at home, and they did not want to disturb their children’s life. However, they were happy to take part in various online activities. Studies have reported that widowed older adults use digital media to increase social opportunities and spiritual support, which can reduce loneliness and social isolation [40, 41]. Especially for the widowed older adults living alone, they can benefit from online social participation and obtain psychological compensation, our findings are consistent with other studies [42, 43]. Finally, online social participation helps enhance older adults’ social connection considerably. Other studies have shown that social networking was a common online activity for older adults [17, 44]. Wilder found that widowed older adults could enhance adaptability to widowhood through frequently contacting with family and friends [45]. Additionally, when the present study’s participants encountered difficulties in using digital media, supports from family and friends further strengthened their connection with their relatives and peers.

Our study shows that the purposes of online social participation for Chinese older adults is relatively simple, focussing on seeking spiritual satisfaction, for example, social networking, entertainment, and information acquisition. This is also in line with a previous study that states that social motivation, entertainment motivation, and information acquisition motivation are the main reasons for older adults using digital media [46]. In our study, we found that online activities of older adults also involve shopping and online learning. However, they do not engage in social activities that require wisdom and creativity, such as political participation, consultation, and decision making. This is in line with the reports of other international studies, that social interaction, shopping, and health-related activities are the predominant activities of online social participation for older adults [19, 47]. In short, the overall level of participation of the older adults in Chongqing is relatively low, focussing on ‘survival’ primary social participation, and ‘developmental’ social participation that requires knowledge, experience, and skills remains insufficient. In this study, the participants were generally found to be concerned about economic losses. It was reported that privacy and security were the most important concern for the older adults [48]. The current network security situation remains severe. Preventing privacy leakages and online frauds are the focus of online activities among the older adults. Moreover, this study shows that older adults are troubled and confused by diverse online activities because of their poor eyesight and limited energy. Meshi revealed that excessive use of social media may also limit the amount of time available, thereby preventing individuals from engaging in social interaction with others offline [49]. Finally, some older adults have difficulties in using the digital media, mainly because they have poor perception and cognition, which is consistent with the findings of Gell’s study [50]. Helsper and Reisdorf found that the older adults lack interest in online activities due to insufficient digital skills [51]. In general, these barriers pose challenges for widowed older adults for participating in online activities.

According to our findings, we speculated that the main needs of widowed older adults for online social participation include emotional needs, digital skill support, and a secure network environment (Fig. 1). Most older adults experience loneliness after being widowed [43]; thus, the emotional needs may be more prominent in this group. Additionally, as online social participation mainly relies on new digital media and the Internet, older adults may attach importance to mastering digital skills and having a secure network environment. Consequently, four suggestions were proposed to improve the social engagement of the older adults based on their needs (Fig. 1). First, widowed older adults should increase the use of digital media to explore its advantages. Higher acceptance with digital media indicated a higher usage rate [52]. Second, family and friends need to continuously provide them with support, including digital skill and emotional support. When they encounter difficulties in using digital media, their family and friends should provide timely help. A previous study has reported that supports from adult children was the main way to solve the skill problems in dealing with online activities [53]. Besides, the older adults who were familiar with digital media were more likely to use them [52]. Meanwhile, it is also important for family and friends to have more online interaction with the widowed older adults. Third, healthcare professionals need to conduct digital skill training and safety publicity campaigns to relieve the skill and security concerns of older adults. Studies have shown that group training can reduce the digital skill anxiety of older adults, help them overcome technical fears, and enhance self-confidence [54]. Meanwhile, healthcare professionals can also encourage older adults to participate in online activities that suit them and guide them to take part in ‘developmental’ activities, such as political participation, online learning, knowledge, and experience sharing, so that they can make contributions to the society while retaining their respect and realizing their self-worth. Finally, further improving the network management system and strengthening supervision is suggested to create a safe network.

### Strengths and limitations

This study enabled us to gain a deeper understanding of a less-explored subject - online social participation among widowed older adults. Widowed older adults are considered a vulnerable group and require more attention. Our findings are context-bound to the participants and study setting. The data were particularly rich in content considering that they were obtained through semi-structured and in-depth individual interviews by two experienced researchers. Our study’s trustworthiness was enhanced through a number of measures as described in the methodology section. Furthermore, this study also provides references for other groups of older adults, especially for the older adults with loneliness and social isolation. They can participate activities anytime and anywhere, and experience its benefits. Although this study has some strengths in comparison to the existing literature on social participation of older adults, there are also two limitations. First, the research was conducted among widowed older adults in Chongqing of southwestern China. Some viewpoints may differ from those in other countries because of the differences in social culture and economic development. Second, there was no detailed focus on the time of widowhood (recently widowed or have been widowed in the distant past), which could impact the online social participation of older adults. In future studies, we can thus explore the impact of widowhood time on the social participation of the older adults. However, despite these limitations, we believe that this is a valuable study, which reveals widowed older adults’ unreported experiences of online social participation.

## Conclusions

Social participation of widowed older adults in southwestern China has begun to be integrated into the digital world; however, it remains at an early stage, with the simple purpose of engagement. Older adults may face great challenges in online social participation. Although there are barriers and challenges with such participation, widowed older adults can actually experience its benefits, which can be considered as an important measure to facilitate successful ageing. Online social participation will unequivocally soon become indispensable. Family, friends, and healthcare professionals should pay more attention to widowed older adults’ needs related to online social participation and provide them with adequate support to achieve a meaningful life during old age.

## Data Availability

The datasets used and/or analysed during the current study are available from the corresponding author upon reasonable request.
